# Thoracic and abdominal outgrowths in early pterygotes: a clue to the common ancestor of winged insects?

**DOI:** 10.1038/s42003-023-05568-6

**Published:** 2023-12-12

**Authors:** Jakub Prokop, Kateřina Rosová, Angelika Leipner, Pavel Sroka

**Affiliations:** 1 Department of Zoology, Faculty of Science, Charles University, Viničná 7, 128 00 Praha 2, Czech Republic; 2 Museum Schölerberg, Klaus-Strick-Weg 10, 49082 Osnabrück, Germany; 3 Institute of Entomology, Biology Centre of the Czech Academy of Sciences, Branišovská 31, 370 05 České Budějovice, Czech Republic

**Keywords:** Palaeontology, Entomology

## Abstract

One of the fundamental questions in insect evolution is the origin of their wings and primary function of ancestral wing precursors. Recent phylogenomic and comparative morphological studies broadly support a terrestrial ancestor of pterygotes, but an aquatic or semiaquatic ancestor cannot be ruled out. Here new features of the branchial system of palaeodictyopteran larvae of several different instars of *Katosaxoniapteron brauneri* gen. et sp. nov. (Eugereonoidea) from the late Carboniferous collected at Piesberg (Germany) are described, which consist of delicate dorsolateral and lamellate caudal abdominal gills that support an aquatic or at least semiaquatic lifestyle for these insects. Moreover, the similar form and surface microstructures on the lateral abdominal outgrowths and thoracic wing pads indicate that paired serial outgrowths on segments of both tagmata presumably functioned as ancestral type of gills resembling a protopterygote model. This is consistent with the hypothesis that the wing sheaths of later stage damselfly larvae in hypoxic conditions have a respiratory role similar to abdominal tracheal gills. Hence, the primary function and driving force for the evolution of the precursors of wing pads and their abdominal homologues could be respiration.

## Abstract (web summary)

New palaeodictyopteran larvae show similar form and surface microstructures on lateral abdominal outgrowths and thoracic wing pads, suggesting that paired serial outgrowths on segments of both tagmata functioned as ancestral type of gills, resembling a protopterygote model.

## Introduction

The appearance of wings is one of the major steps in hexapod evolutionary history. The main question about the origin of wings, is what was the initial function of the wing precursors and the part of the body involved in their formation (e.g., Clark-Hachtel and Tomoyasu^1^, Ohde et al.^2^). The proposed function of wing precursors largely depends on whether the ancestor of winged insects was terrestrial or aquatic.

Hasenfuss^3^ provides a broad overview of the fossil evidence for the protopterygote ancestor. One of the hypotheses for the origin of wings is that it is associated with the transition from an aquatic to an aerial life style (aloft from the water surface) and thus the crucial role of aquatic environments^4^. Some other studies consider wings as derived from ancestral gills^5^ and early developmental studies confirm the evolutionary relationship between crustacean gills and the insect tracheal system^6,7^.

Nevertheless, the current scenario widely supports a terrestrial ancestor for pterygotes^8,9^ with terrestrial Zygentoma (silverfish) as a sister group of Pterygota. Wipfler et al.’s^8^ phylogenetic analysis of habitus and lifestyle characters within Polyneoptera (and Pterygota in general) indicates that all of the developmental stages in the ancestors of both these groups were terrestrial and thus wings did not originate from an aquatic ancestor. This conclusion, however, was based on a cladogram only using a dataset of recent species and omitted the enormous record of extinct relatives traceable back at least for 300 mya^10–12^. The origin of the Pterygota and habitat or lifestyles of the ancestor of pterygotes are key questions, which could be better understood when fossils from the Late Paleozoic, particularly those with marked apomorphies/specializations at the super ordinal level such as Palaeodictyopterida, are considered^13,14^. According to Sharma^9^, ancestral state reconstructions must be interpreted critically, particularly when based only on extant groups (terminals) and when the fossil record for a group suggests historical extinctions of higher-level clades. Thus, an aquatic ancestor for Pterygota cannot be definitively excluded. But what evidence could be revealed by studying the early pterygotes recorded in the Carboniferous? Crucial information can be gathered when focusing on their respiratory apparatus.

Respiration, or the transport of oxygen from the environment to tissues and expelling of carbon dioxide in insects is mainly via an air-filled tracheal system. Usually, it is open to the environment and air passes into the tracheae through circular openings in the body-wall, the spiracles. In some aquatic insects, however, the tracheal system is closed to the environment and gas diffuses directly through the thin integument into the tracheal system^15^. In these insects the respiratory surface is often enlarged for maximal diffusion by various types of tracheal gills, which originated independently in different lineages and occur on various body parts, such as mouthparts, adhered to proximal podomeres or between abdominal segments. Aquatic or semiaquatic larvae with abdominal tracheal gills occur in both extant lineages of the clade Palaeoptera, comprising dragonflies (Odonata) and mayflies (Ephemeroptera). Their stem groups and earliest representatives recorded since the late Carboniferous or early Permian are also characterized by aquatic larvae^16,17^. Though there is a Paleozoic record of aquatic immatures for several polyneopteran lineages, like the stem group of Plecoptera or some enigmatic “Grylloblattida” (Lemmatophoridae), their higher phylogenetic relationships, especially in respect of the crown group of Plecoptera remain unresolved^10,18,19^.

The superorder Palaeodictyopterida with specialized piercing and sucking mouthparts in the form of a rostrum is among the first recorded pterygotes and its phylogenetic placement as stem group of Neoptera is resolved^13,14,20^. Specializations for aquatic or semiaquatic lifestyles in larvae of Palaeodictyoptera have been studied for more than a century, but the interpretations differ greatly^21–25^. Caudal appendages of palaeodictyopteran larvae with a presumed respiratory function were recently reported in an early instar of *Idoptilus* sp.^26^, whereas in all others only cerci are documented^27,28^ and exceptionally interpreted as long ovipositors^29^.

Here is presented a series of larval exuviae of different ontogenetic stages of a palaeodictyopteran attributed to *Katosaxoniapteron brauneri* gen. et sp. nov. (Eugereonoidea), which were recently discovered in Pennsylvanian deposits at Piesberg quarry (Lower Saxony, Germany). The examination of this series using Environmental scanning electron microscopy (ESEM) revealed, in remarkable detail, the structure of thoracic and abdominal outgrowths. This provides the first detailed information on the structure and especially a possible function of abdominal lateral and caudal outgrowths in these Paleozoic insects, which are currently accepted as the stem-group of Neoptera.

## Results

### Systematic palaeontology

Order Palaeodictyoptera

Superfamily Eugereonoidea

Family uncertain

Genus *Katosaxoniapteron* gen. nov.

### Type species

*Katosaxoniapteron brauneri* gen. et sp. nov. by present description

### Etymology

Composite name consisting of the region Lower Saxony (Katosaxonia in Greek) and *pteron* for wing in Ancient Greek.

### Diagnosis

Larval characters: Long and cylindrical body with prominent prothoracic lobes, meso- and metathoracic wing pads, and flattened abdominal lateral outgrowths (flaps) on segments I–IX with numerous filaments (papillae) that often project beyond the outer edges. Caudal appendages with enlarged lamellar paraprocts.

Forewing pads with prominent anterior keel in costal area, developing pattern of venation with concave ScP diverging from C (towards RA distally), three precursors of the convex simple veins RA, MA and CuA, and more extensively branched RP, MP and CuP. Precursor of convex vein PCu clearly discernible, anal area relatively broad with numerous branches. Metathoracic wing pads with smaller costal area. Abdomen heavily sclerotized, segments I–IX bear a pair of hinged cordate lateral outgrowths broadly fused to tergum. Abdominal segment XI in form of caudal appendages, which consist of paired long cone-shaped lateral cerci secondarily multijointed and a pair of ventral lamellar paraprocts obliquely directed backwards.

*Katosaxoniapteron brauneri* gen. nov. et sp. nov.

(Figs. 1–4).

**Fig. 1:**
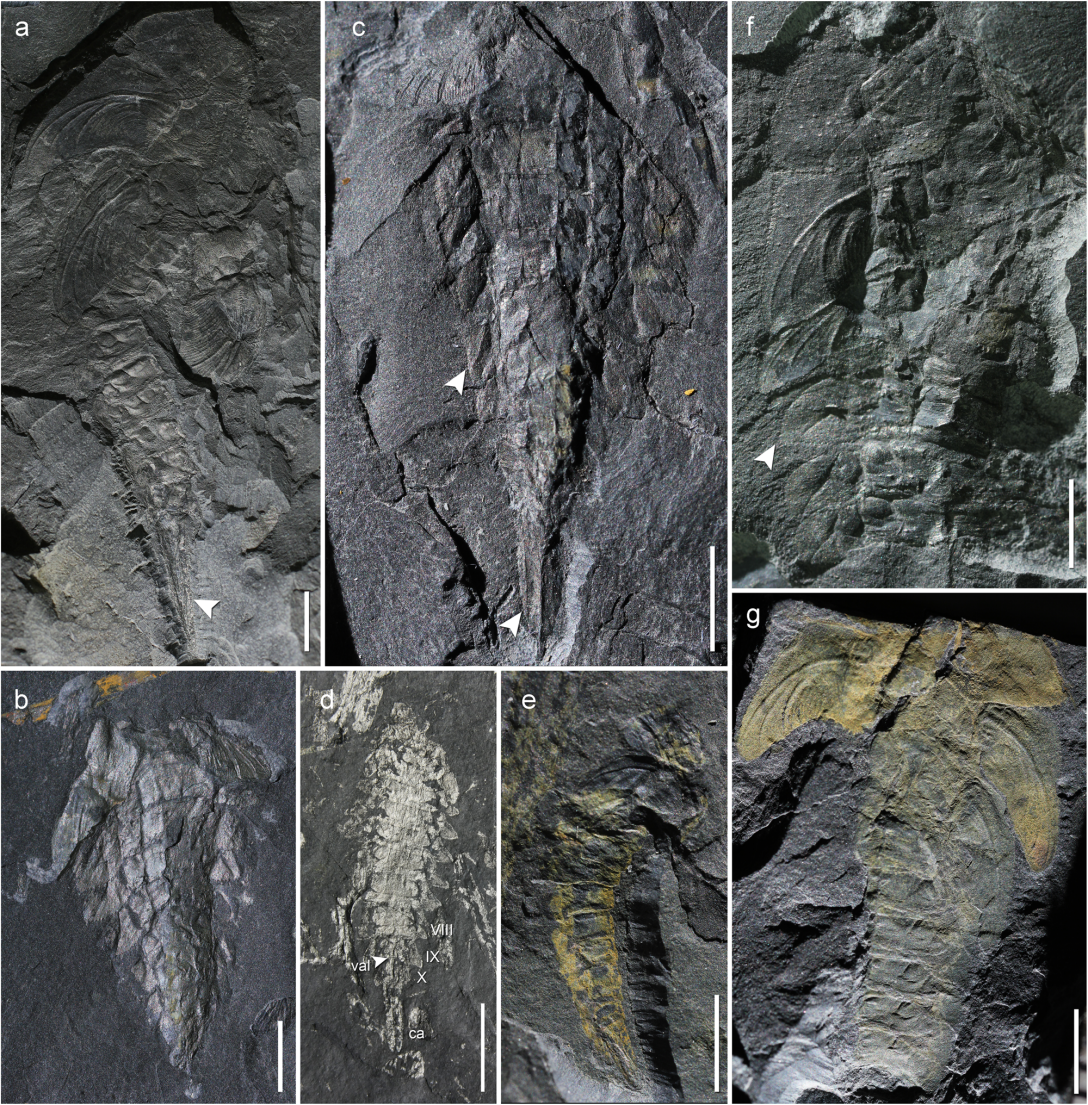
Larval exuviae of various instars of *Katosaxoniapteron brauneri* gen. nov. et sp. nov. (Eugereonoidea), a palaeodictyopteran from Moscovian deposits at Piesberg. **a** Late (mature) instar, Holotype Pal1242, habitus in dorsolateral view. **b**, **c** ?Exuviae of an early instar larva, No. F148, habitus in dorsal view. **d** Larval exuvia Pal cl 4, habitus in ventral view showing abdominal lateral outgrowths, valvular ovipositor (val) and caudal appendages (ca). **e** Early instar larva Pal1243, habitus in dorsolateral view. **f** Middle instar larva F375, habitus in dorsal view. **g** Larval exuvia F139, habitus in dorsal view. White arrows show lateral and caudal abdominal outgrowths. Scale bars are **a**, **d** = 5 mm; **b**, **c**, **e**–**g** = 3 mm.

**Fig. 2:**
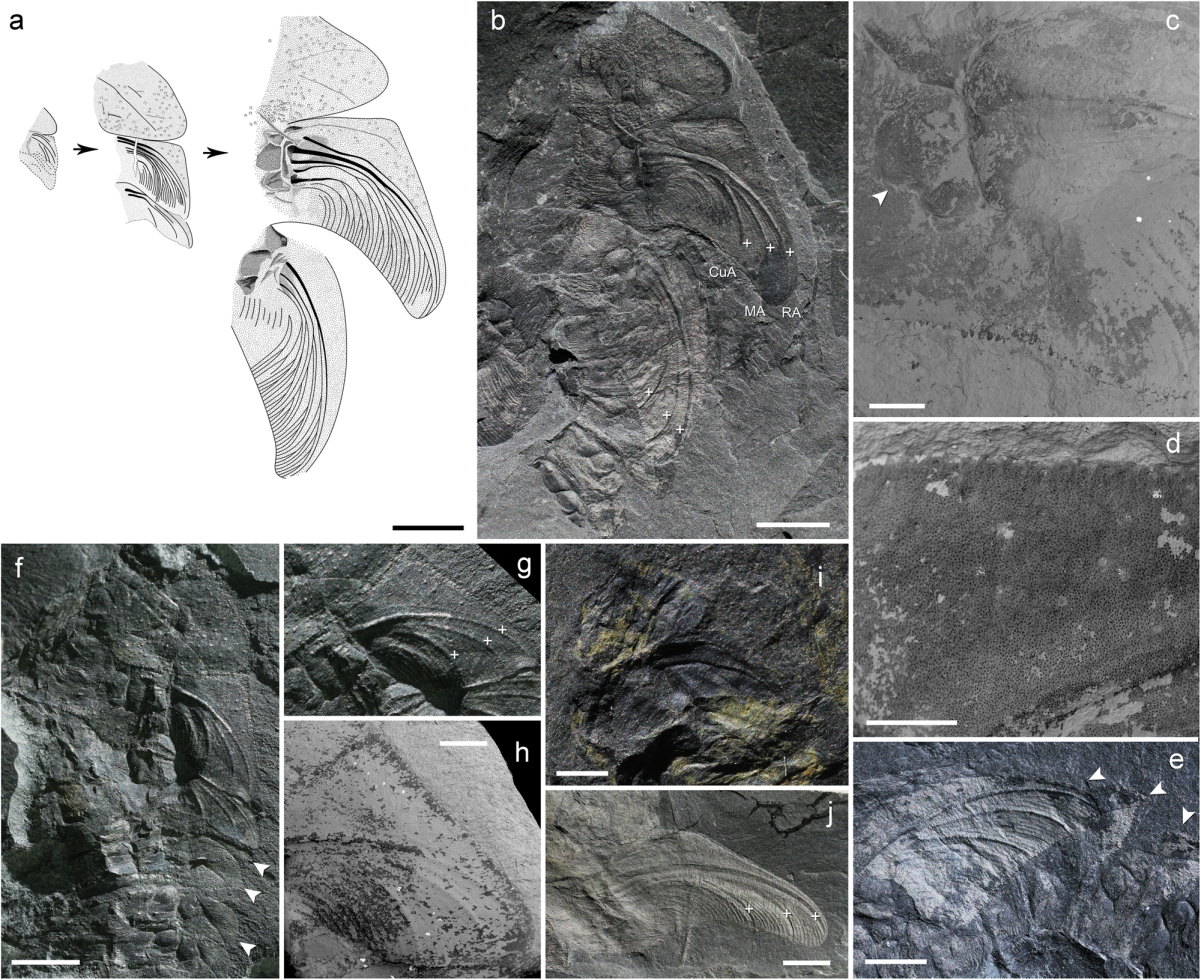
Prothoracic lobes and meso- and metathoracic wing pads of exuviae of larvae of *Katosaxoniapteron brauneri* gen. nov. et sp. nov. (Eugereonoidea) from Moscovian deposits at Piesberg. **a** Line drawings of developing wings in sheaths showing the precursors of future veins in specimens (from left to right: based on specimens Nos. Pal1243, F375 and Pal1242). **b**–**e** Pal1242, detail of prothoracic lobe and wing pads of holotype, **c** developing articulation of wing pads, **d** detail of structure of keel in costal area and anterior margin of wing pad, **e** structure of metathoracic wing pad and lateral abdominal outgrowths on segments III and IV. **f**–**h** F375, **f** detail of prothoracic lobe, wing pads and proximal abdominal lateral outgrowths, **g** structure of mesothoracic wing pad with precursors of veins and prominent anterior keel, **h** micrograph of mesothoracic wing pad. **i** Pal1243, detail of wing pads and proximal abdominal segments. **j** Pal1246 mesothoracic wing pad. Precursors of future veins with convex lacunae indicated by plus. Scale bars are **a**, **b** = 5 mm; **c**, **d** = 1 mm; **f**, **h**, **j** = 3 mm.

**Fig. 3:**
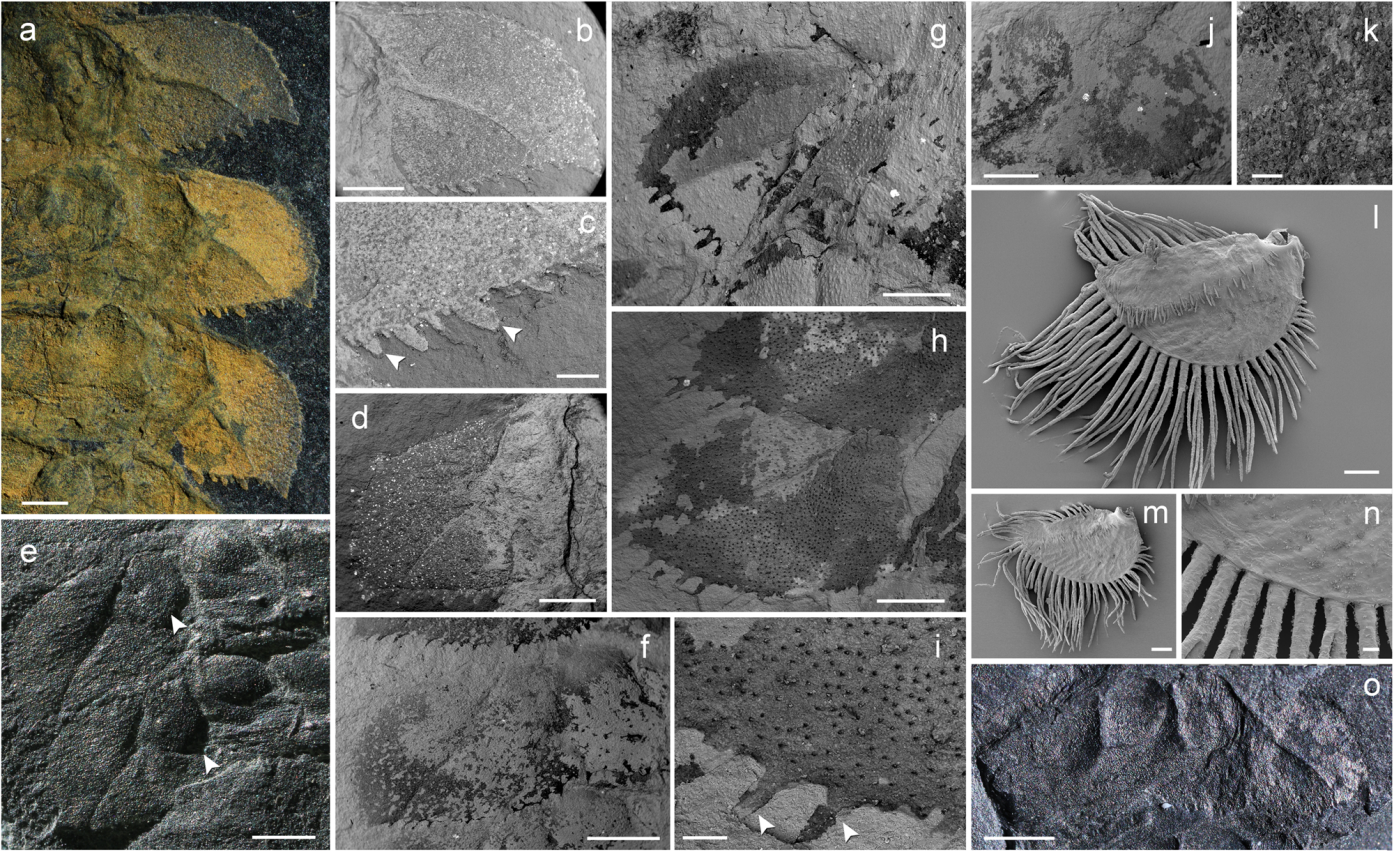
Abdomen with dorsolateral outgrowths of larvae of *Katosaxoniapteron brauneri* gen. nov. et sp. nov. (Palaeodictyoptera: Eugereonoidea) from Moscovian deposits at Piesberg and comparative extant material. **a**–**d** F 429, photographs and SEM micrographs of abdominal dorsolateral outgrowths (flaps) with distinct papillae. **e**, **f** F375, photograph and SEM micrograph of abdominal dorsolateral outgrowths (flaps) with articulation and distinct papillae. **g**–**i** F148, micrographs of dorsolateral outgrowths. **j**, **k**, **o** Pal1242, photograph and SEM micrographs of detail of dorsolateral outgrowths (flaps). **l**–**n** *Caenis* sp. (Caenidae), micrographs of abdominal tracheal gill in form of a plate-like structure with fringed margin. Scale bars are **a**, **b**, **d**–**f**, **j**, **o** = 1 mm, **n** = 10 µm; **i**, **k**, **l**, **m** = 100 µm; **c** = 300 µm; **g**, **h** = 500 µm.

**Fig. 4:**
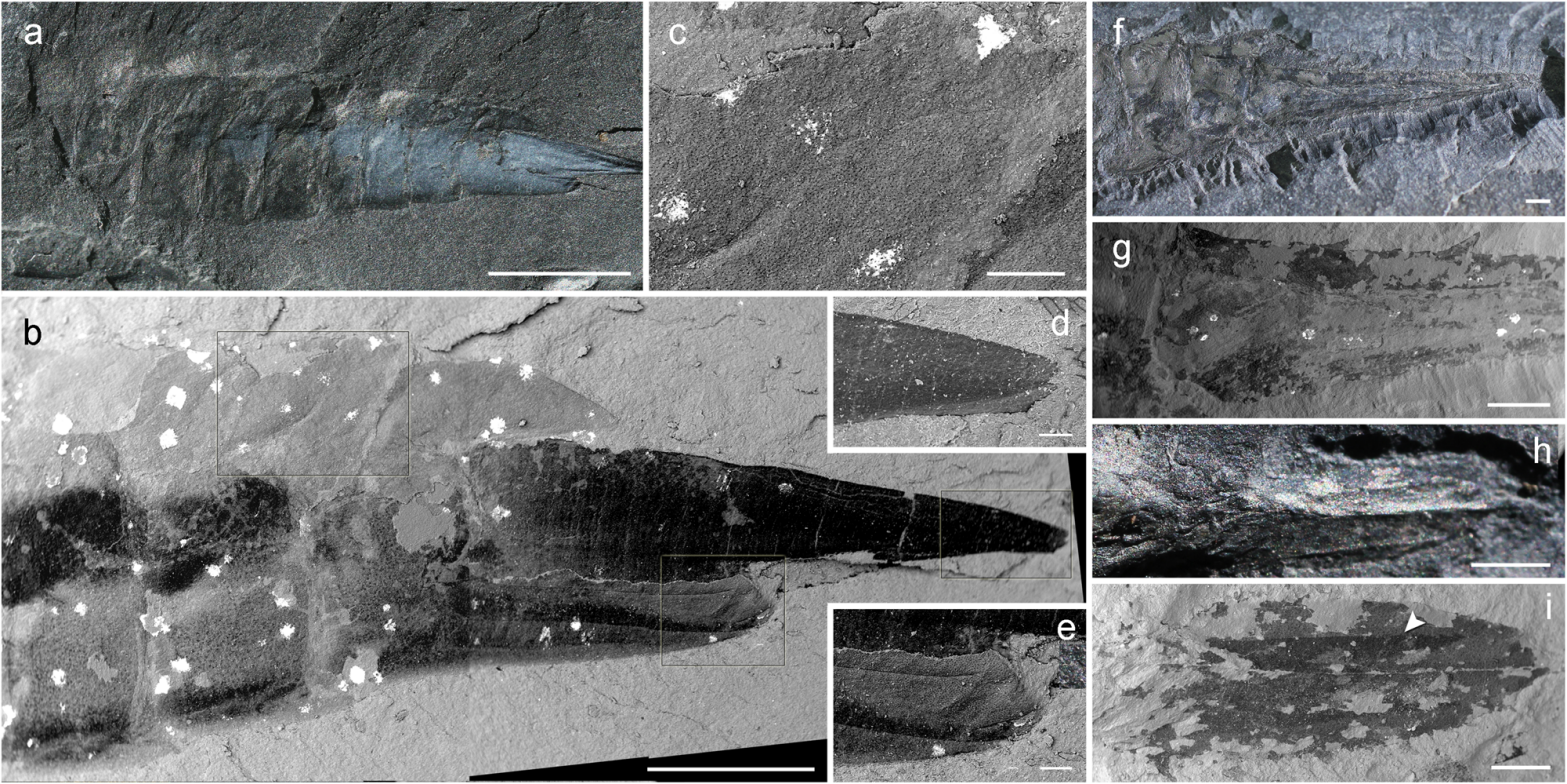
Abdomen with caudal outgrowths of larvae of *Katosaxoniapteron brauneri* gen. nov. et sp. nov. (Palaeodictyoptera: Eugereonoidea) from Moscovian deposits at Piesberg. **a**–**e** F427, apical part of abdomen with dorsolateral and caudal outgrowths. **f**, **g** Pal1242, photograph and SEM micrograph of caudal outgrowths. **h**, **i** F148, photograph and SEM micrograph of caudal  outgrowths. Scale bars are **a** = 3 mm; **b**, **f**, **g**, **h** = 1 mm, **c** = 150 µm, **d** = 90 µm, **e** = 120 µm, **i** = 500 µm.

### Etymology

The specific epithet named after Stephan Brauner, who collected the holotype and one of the supplementary specimens (Pal 1243).

### Holotype

Pal1242ab (claystone above coal seam Dreibänke) was previously considered to be a nymph of “Rochdalia type,” with an ovipositor; presumed to be exuvia due to the distortion of metathoracic wing pads^29^; prothoracic winglet lobe without a distinct articulation area—lacunae very faint, meso- and metathoracic wing pads with well discernible pattern of lacunae and abdomen with terminalia.

### Additional material

Pal1243, F429ab, F139, F148ab, F246ab, F320, F375ab, F427ab; Pal cl 4; for details, see Supplementary Note 1.

### Description

Habitus largely based on larval exuviae that have a number of shared traits and presumably correspond to different ontogenetic stages (instars), as documented by the varying pattern of the vein precursors on wing pads and body length. Cephalic structures not preserved, prothorax bears well developed triangular prothoracic lobes with faint traces of basal articulation and single discernible precursor of a concave vein (lacuna) oriented towards the posterior outer edge of lobe (Pal1242, F375, F139), mesothorax larger than prothorax with clearly developing articular joint of mesothoracic wing pads consisting of axillary sclerites (sensu Hamilton^30^, Prokop et al.^31^) and broadly fused posteriorly, forewing pads in perpendicular position relative to the body axis showing prominent anterior keel in costal area, enlarged outer margin typical for wing sheaths, developing pattern of venation with concave ScP diverging from C (towards RA distally), three precursors of the convex simple veins RA, MA and CuA, and more extensively branched RP, MP and CuP (Pal1242, F246, F148, F375) varying in number of branches depending on body size (see Supplementary Table 1). Precursor of convex vein PCu clearly discernible (Pal1242, F139), anal area relatively broad with numerous branches. Metathoracic wing pads resemble in size and developing pattern of venation those on the mesothorax, but with smaller costal areas. Their original position relative to the body axis is probably largely affected by distortion of the cuticle during moulting (Pal1242). Abdomen of clearly 10 segments with heavily sclerotized terga and segment XI in form of caudal appendages (Pal1242, F427), which consist of paired long cone-shaped lateral cerci secondarily multijointed (F427) and a pair of ventral lamellar paraprocts obliquely directed backwards (Pal1242, F320, F148, F427). Abdominal segments I-IX bear a pair of hinged cordate lateral outgrowths broadly fused to tergum (F429, F148). Female ovipositor in form of two pairs of cutting valvulae and a pair of sheathing valvulae that appear to originate ventrally from segments VIII and IX (Pal cl 4).

### Dimensions

For length and width of forewing pads, estimated length of thorax plus abdomen excluding caudal appendages, see Supplementary Table 1.

### Systematic position

The larval exuviae of *Katosaxoniapteron brauneri* gen. nov. et sp. nov. can be attributed to the order Palaeodictyoptera based on the form and structure of the wing pads as well as characteristic abdominal outgrowths, although the distinctive haustellate mouthparts are not preserved on any specimen (Prokop et al.^25^). Palaeodictyopteran larvae are recognizable by large keel in the costal area on the forewings and markedly pleated venation with well-developed convex precursor of vein MA^27,32^. Moreover, the prothorax in some palaeodictyopterans bears protruding triangular lateral lobes resembling the meso- and metathoracic wing pads, which are also present in this species. The larvae of palaeodictyopterans like other exopterygotes have exposed wing pads with their developing wing venation in the form of lacunae based on which it is possible to tentatively infer their systematic position and association with adults of a particular superfamily or family, and compare them with other described larvae or their exuviae. Based on the pattern of lacunae of the precursor of veins RA, MA and CuA, these larvae are placed in the superfamily Eugereonoidea, currently comprising 12 families^33^. Adult representatives of the three families: Archaemegaptilidae, Lithomanteidae, and Eugeneroidae in the Eugereonoidea are recorded from the Piesberg quarry and descriptions of new species attributable to Archaemegaptilidae are currently under preparation^34–36^. In addition to the characteristic precursors of the veins RA, MA and CuA in all the specimens studied, there are numerous branches of RP and MP, which are more extensively developed in supposedly older instar larvae (Pal1242) and less branched in earlier instars (F148, F246, F375, Pal1243). Hence, the number of posterior branches of the RP and MP veins increases as the larva increases in size and wing pads enlarge (see Fig. 2a and Supplementary Table 1).

**Fig. 5:**
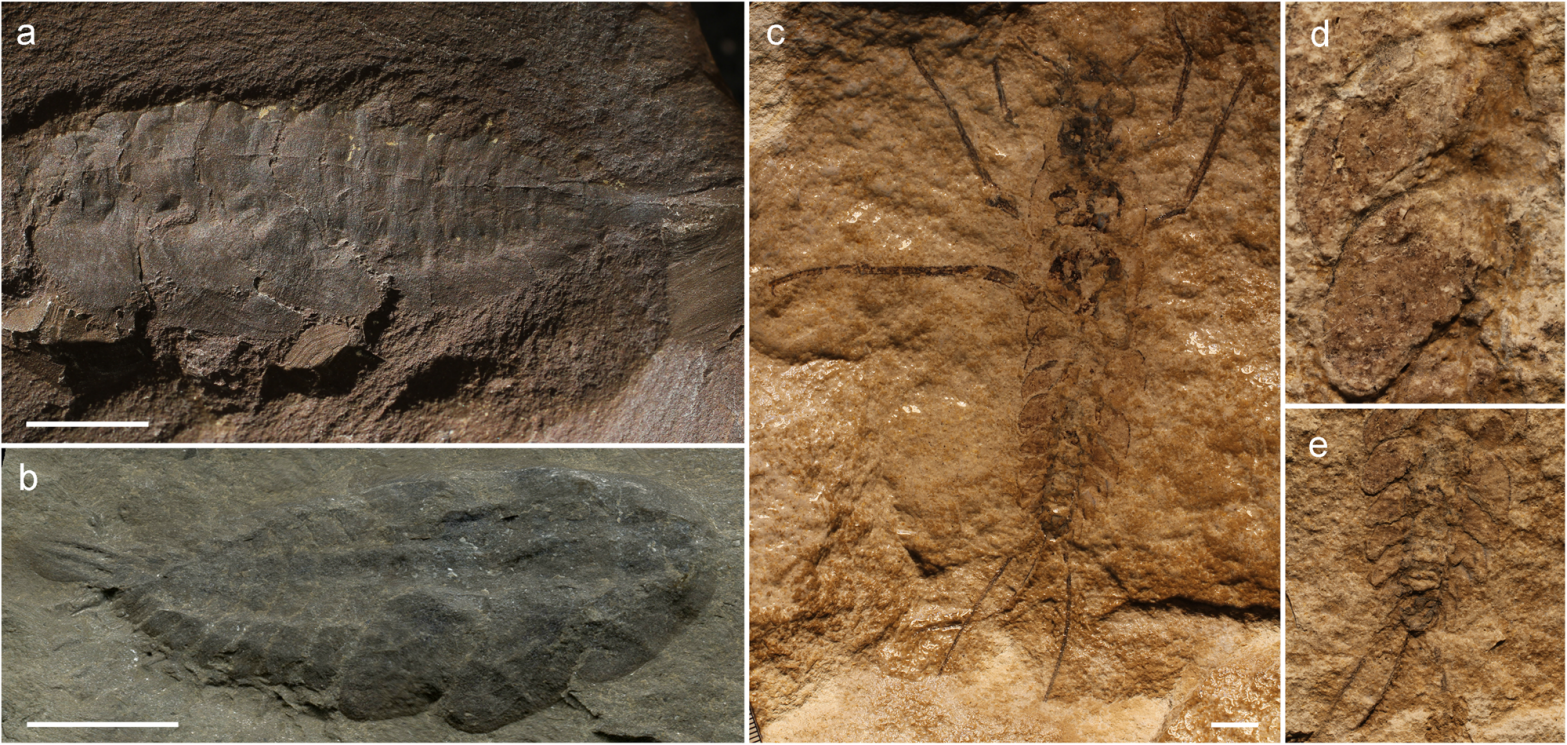
Habitus, thoracic and abdominal outgrowths of larvae of Palaeodictyoptera and Carbotriplurida. **a** *Rochdalia parkeri* (Palaeodictyoptera), habitus in dorsolateral view, No. MM L.11464 Manchester Museum coll., UK; **b** *Idoptilus* sp. (Palaeodictyoptera), habitus in dorsolateral view, GLAHM A.2680a Hunterian Museum, University of Glasgow, UK coll.; **c**–**e** *Carbotriplura kukalovae*, habitus in dorsal view, Museum of Czech Karst coll. Beroun, Czech Republic; **d** detail of thoracic lateral outgrowths, **e** detail of abdomen with lateral outgrowths. Scale bars are **a**, **b** = 5 mm; **c** = 10 mm.

Part of this material was previously studied by Kiesmüller et al.^29^; however, they only used light stereomicroscopy to study the long caudal outgrowths and did not carry out a detailed examination of the structure of the wing pads. Their study considered the material to be associated with the palaeodictyopteran *Rochdalia* Woodward, 1913 known from the Sparth Bottoms (Lower Coal Measures) in Rochdale (Lancashire, UK)^37^. Unfortunately details of the pattern of vein precursors on wing pads of the holotype *Rochdalia parkeri* Woodward, 1913 are not clearly discernible (see Fig. 5a) and therefore it is difficult to compare it with this material^27^. The convex lacunae, however, are apically very close to each other in the corresponding instar (F375), so the exact number of terminal branches of RP is lower than in *Katosaxoniapteron brauneri* gen. et sp. nov. We concur with Kiesmüller et al.^29^ that all larval specimens from the Piesberg quarry are probably conspecific and represent different ontogenetic stages (instars) with presumably more extensively developed concave branches of RP and MP in later instars (see Fig. 2a).

## Discussion

The crucial issue for the functional interpretation of Palaeodictyoptera larvae from Piesberg is whether they were terrestrial or aquatic. It is not always easy to distinguish between aquatic and terrestrial fossil larvae. The strongest evidence is the presence of aquatic adaptations, usually related to locomotion or respiration^38^, often the presence of gills of a specific type. Aquatic larvae breathe through their cuticle and gills represent enlarged well-tracheated cuticular surfaces that increase the capacity for oxygen uptake in a freshwater environment. Gills can be of many different forms and located on different parts of the body. They occur on cephalic structures such as mouthparts, on legs and most commonly as tracheal gills inserted posterolaterally on abdominal segments. This arrangement evolved several times independently in various lineages of insects, like mayflies (Ephemeroptera), dragonflies and damselflies (Odonata), stoneflies (Plecoptera), alderflies (Megaloptera), lacewings (Neuroptera), some groups of beetles (Coleoptera), and others^15,39^.

In the Piesberg palaeodictyopteran larvae of *Katosaxoniapteron brauneri* gen. et sp. nov., such typical abdominal gills with a posterolateral articulation point, typical of mayflies, are not present. However, pronounced lateral outgrowths on abdominal segments (so called flaps) might have served the same purpose. In larvae of Palaeodictyoptera, these outgrowths are considered to be paratergal lobes by previous authors^27,29^ or as derived entirely from pleural epicoxa^24,40^. Detailed examination revealed an anteromedial articulation and an anterior and broad posterior fusion in larvae described from Sosnowiec in Poland indicating their homology with developing wings^41^. The similar form and articulation in larvae of *K. brauneri* from Piesberg (Fig. 1f), indicate limited mobility. The detailed structure on the surface of these lateral outgrowths visualized using ESEM indicate numerous filaments (papillae) that often project beyond the outer edges of these cordial flaps (see Fig. 3a–c, i). These filaments are definitely not spine-like setae or similar articulated structures as they are not pointed or more sclerotized and they lack articulation (see Fig. 3c, i). They form an integral part of the hinged outgrowth (flap or “paratergum”). This arrangement is reminiscent of the filamentous plate-like gills in some Recent mayflies, like the families Leptophlebiidae and Caenidae, in which the tracheal gills consist of plate-like structures with fringed margins (e.g., Merritt and Cummins^42^ Figs. 8.15 and 8.27) and possibly have a similar function (see Fig. 3l–n). However, the filaments of *K. brauneri* are relatively shorter compared to those found in extant mayfly larvae, thus, the role of these filaments in respiration was probably not as crucial. Still, even short filaments increased the surface area of the flap and could aid respiration.

Interestingly the abdominal tracheal gills of Ephemeroptera are considered to be possible homologs of wings by several authors and recent studies revealed both structures share a common genetic program (e.g., Wigglesworth^43^, Kluge^44^, Almudi et al.^45^). However, the origin of these structures remains unexplained in respect of the participation of tergal or pleural tissues^1^ and some authors suggest that wings evolved from the pre-existing lateral terga of a wingless insect ancestor^2^.

The structure of the caudal appendages of palaeodictyopteran larvae from Piesberg also provide evidence of an aquatic lifestyle. They are in the form of paired cone-shaped stout lateral cerci and vertically oblique paired ventral paraprocts, which are similar to the lamellate caudal appendages of some modern zygopteran larvae (Odonata). Scattered long spines are discernible on the obliquely directed paraprocts (Fig. 4b, e). Although in some odonatan larvae caudal appendages greatly change in form and presumably function during ontogeny (Norling^46^: Fig. 1g, Rowe^47^: pp. 352–354), lamellate appendages are generally used for breathing (Tillyard^48,49^: Text figs. 20–29, Corbet^50^: Figs. 4.5–4.7). Shape and posture of lamellate paraprocts correlate with habitat and availability of dissolved oxygen, for example, vertically held lamellae occur in species inhabiting poorly oxygenated habitats, i.e., standing water^50^. Similarly, the vertically held paraprocts in the Piesberg larvae might indicate a similar type of habitat.

Caudal appendages of Piesberg larvae were previously interpreted differently. Kiesmüller et al.^29^ consider these structures to be parts of an exposed ovipositor. However, based on previous studies of the female genitalia with an external valvular ovipositor in Palaeodictyoptera it consists of three pairs of valvulae derived from abdominal segments VIII and IX (gonapophyses), with two pairs of cutting valvulae and a pair of sheathing valvulae (gonoplacs of segment IX). Except for female imagoes, external ovipositors are also documented in several palaeodictyopteran larvae, like *Paimbia* cf. *fenestrata* Sinitshenkova^51^ and *Bizarrea obscura* Prokop et al.^28^. Neither of these correspond with the pattern in Piesberg larvae of *K. brauneri*, where caudal appendages originate posteriorly from segment X (Figs. 1a–c and 4a, b, f–i). Therefore, the putative long ovipositors described by Kiesmüller et al.^29^ are caudal appendages derived from terminal segment XI (see above). Moreover, in one of the newly discovered larvae at Piesberg (Pal cl 4), a true ovipositor is clearly visible, which is different from the caudal appendages discussed above (Fig. 1d).

Within Palaeodictyoptera, lamellate paraprocts indicating an aquatic lifestyle were first reported in early stage larval exuviae of *Idoptilus* sp. from Bolsovian of Stainborough, Barnsley (Middle Coal Measures) in South Yorkshire, UK^26^ (Fig. 5b). A detailed re-examination of the caudal appendages of a larva of another species, *R. parkeri*, revealed the presence of oblique ventral paraprocts along the stout annulated cerci.

The enlarged paraprocts of larvae can be retained to some extent also in imagoes. Re-examination of several specimens with completely preserved abdomens revealed vestigial paraprocts. Surprisingly, similar caudal abdominal appendages were first described already by Brongniart^21^ in a female imago of *Lycocercus goldenbergi* (Lycocercidae) preserved in lateral aspect and interpreted as dorsal appendages “crochets dorsaux.” Kukalová^52^ ^(p. 454)^ suggests that these are connected at the base to cerci and are remnants of the moulted cuticle of the cutting valvulae of the ovipositor. Although these appendages are orientated vertically, they could be displaced vestigial ventral paraprocts. This would point to the existence of lamellar paraprocts also in the larvae of *L. goldenbergi*, which were possibly used for aquatic respiration.

In addition to the abdominal lateral and caudal structures, it is possible that Piesberg palaeodictyopteran larvae also used their wing pads for respiration. The form of the wing pads in *K. brauneri* is remarkably similar to the abdominal lateral outgrowths in terms of surface microstructure and point of articulation, particularly in older instar larvae (see Figs. 1f and 2e, f). This is consistent with the behavior of later stage damselfly larvae in hypoxic conditions when the wing sheaths have a respiratory role similar to abdominal tracheal gills. Later stages of zygopteran larvae in oxygen deficient conditions can spread their wing pads laterally by about 15–20° for better exposure^50,53^. This limited wing pad movability may account for the slightly different angle of wing pads to body axis in individual exuviae of *K. brauneri* from Piesberg. However, it might also result from the distortion of the thoracic cuticle during moulting.

In summary, the larvae of *Katosaxoniapteron brauneri* gen. nov. et sp. nov. (Palaeodictyoptera) from Piesberg were aquatic or at least semiaquatic. This is based on the presence of cuticular respiratory surfaces in the form of pronounced flattened and marginally fringed abdominal lateral outgrowths (flaps), enlarged lamellar paraprocts, a pair of prothoracic lobes and two pairs of wing pads, possibly all used for oxygen uptake. It remains questionable how widespread the aquatic lifestyle was within Palaeodictyoptera as the fossils of their larvae are extremely rare. Further support for an aquatic lifestyle is the retention of vestigial gill like structures in adults, which are also recorded in other lineages of Palaeodictyopterida, such as Megasecoptera^25^. Hence, it could represent the ancestral state within this clade.

The fact that aquatic specializations occur in Palaeodictyoptera, an early group of Pterygota, supports the earlier hypothesis that the immature stages of protopterygote were aquatic (e.g., Brauer^54^, Handlirsch^22^, Shear and Kukalová-Peck^55^, Kingsolver and Koehl^56^). However, the early evolution of pterygotes is still an open issue due to uncertain monophyly of Palaeoptera and the obscured origin of insect flight^57^. The Recent sister group of Pterygota is represented by silverfish (Zygentoma), which are exclusively terrestrial, although little is known about their early evolution (the oldest Zygentoma fossils date only to the Lower Cretaceous see Rasnitsyn^58^). However, considering the Late Paleozoic hexapod record it is not possible to exclude that the transitional fossil link between Zygentoma and Pterygota, *Carbotriplura kukalovae* was either aquatic or semiaquatic. This insect had prominent lateral tergal outgrowths on the thoracic and abdominal segments, and possibly used these plate-like structures for respiration as precursors of gills^59^ (see Fig. 5c–e), although no other supportive aquatic adaptations are documented^60^.

The laterally positioned and dorsoventrally flattened wing precursors might have originally served for respiration in the larval stage, only later being retained in the adult stage. It is not exceptional that various gill like structures of aquatic larvae are retained even in terrestrial adults^61,62^. In this scenario, the persistence of larval thoracic paraterga in adults could have resulted in them being used for gliding and the start of the evolution of powered flight^3,63,64^.

However, the primary selective pressure for the development of thin lateral outgrowths could have been it increased the respiratory surface in the larval stage. It could have provided a selective advantage even when such outgrowths were relatively small and ineffective for gliding. This view is also compatible with a rowing and surface-skimming locomotion hypothesis for extracting oxygen from water by gill movement, which is reported for some extant stoneflies^65,66^. Interestingly, recent studies report the presence of tracheae in the wing membrane of the adult libellulid dragonfly *Zenithoptera lanei* (Odonata), which have an uncertain role but indicate they could also have been retained by adults of ancient pterygotes^67^.

We speculate that the development of more efficient respiratory devices such as fully articulated abdominal tracheal gills in some groups, substituted the role of wing precursors for respiration. This resulted in these precursors being positioned more posteriorly, partially fused with the notum and less exposed. At that stage of evolution, the function of wing precursors would have been already centered as devices helping gliding in the adult, their original respiratory function being suppressed. A recent study on the developmental origin and growth of wings in the cricket *Gryllus bimaculatus* found that the lateral tergal margin is homologous in primary wingless insects and pterygote insects and consists of a growth organizer for expanding the body wall to form adult wing blades^2^. Hence the precursors of wings and presumably also abdominal dorso-lateral outgrowths (flaps) evolved from pre-existing lateral terga of a wingless insect ancestor with presumed subsequent articulation. This scenario is compatible with the evidence presented for the various ontogenetic stages of the lateral outgrowths of *Katosaxoniapteron brauneri*, where the terga of early instar larvae only have lateral expansions whereas mature larvae have outgrowths (flaps) with distinct articulations. The postponed development of wing pads and abdominal gills to later instars in many extant aquatic groups is in accordance with this idea (reflects the increased oxygen demands of mature larvae and increase in the surface area of their body).

**Fig. 6:**
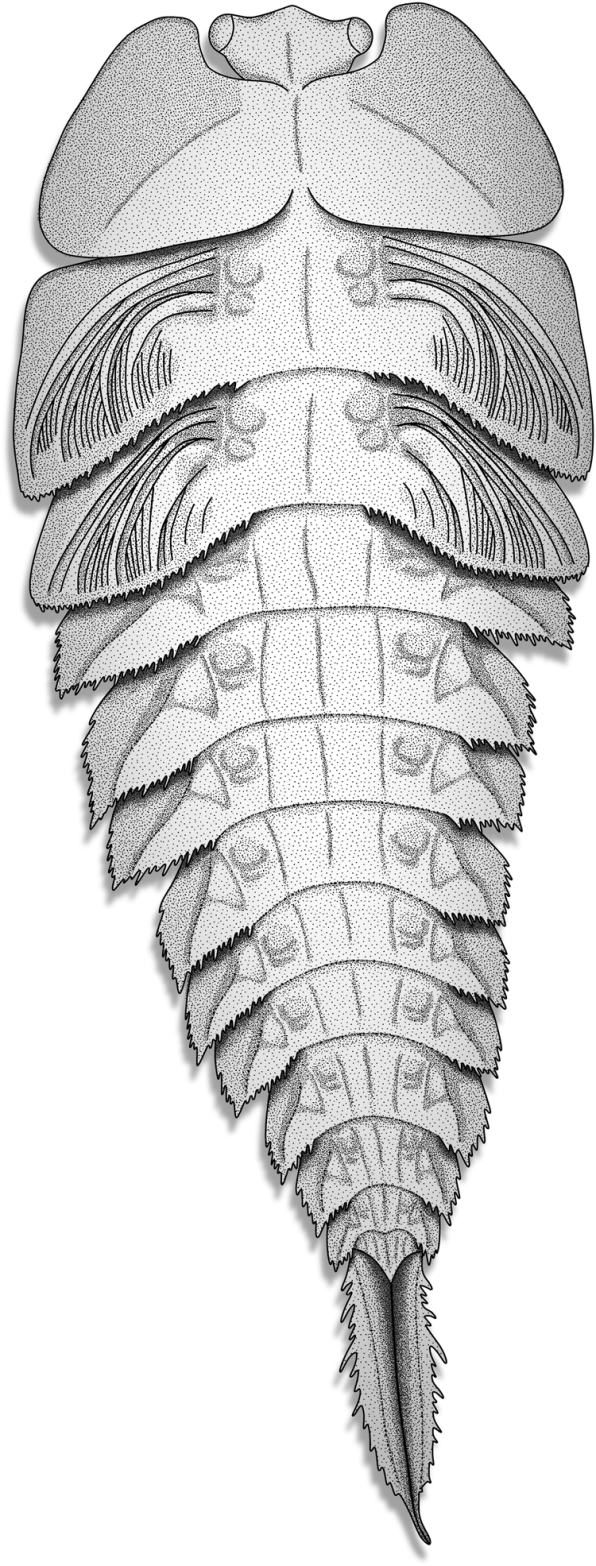
*Katosaxoniapteron brauneri* gen. nov. et sp. nov. (Palaeodictyoptera: Eugereonoidea), middle instar larva, reconstruction of habitus in dorsal view mainly based on specimens Nos. F148 and F375. This habitus is reminiscent of a protopterygote with long cylindrical body and comparable thoracic and abdominal movable lateral outgrowths (flaps). Estimated body length without terminalia 30 mm (F148). (reconstruction drawn by K.R.).

To conclude, the long and cylindrical body of the palaeodictyopteran larvae of *Katosaxoniapteron brauneri* gen. nov. et sp. nov. (Fig. 6) superficially resembles *Protereisma* sp. (Permoplectoptera), the Permian stem group of Ephemeroptera in terms of the serial organization of thoracic and abdominal dorso-lateral outgrowths as wing pads and gills^68,69^. Nevertheless, the tracheal gills in Permoplectoptera are inserted posterolaterally in contrast to *K. brauneri* and other palaeodictyopteran larvae^17,41^. The larvae of *K. brauneri* have prominent lateral outgrowths on their abdomen similar to plate-like gills with respiratory structures like filamentous papillae (fringed margin), which support their having an aquatic or semiaquatic lifestyle. This is further supported by the presence of specialized caudal abdominal appendages in the form of nearly vertically held lamellate paraprocts as in damselfly larvae of the group Odonata (Zygoptera) inhabiting various aquatic or semiaquatic environments^50^. Based on their remarkably similar form and structure the wing pads and abdominal lateral outgrowths (flaps) are considered to be homologous and perhaps one step closer to the protopterygote (ancestor of Pterygota) and provide strong support for the idea that wings evolved from precursors of ancestral gills^5^.

## Methods

### Material

*Katosaxoniapteron brauneri* (Pal1242ab, Pal1243, F429ab, F139, F148ab, F246ab, F320, F375ab, F427ab, Pal cl 4): All specimens were collected from Osnabrück Formation, Clay stone layer above the Dreibänke coal seam, except Pal cl 4, which was collected from the lake sediment between coal seam Mittel and Johannisstein without underlying coal seam, Piesberg Quarry near Osnabrück, Lower Saxony, Germany, Late Carboniferous (Pennsylvanian: Moscovian - Westphalian D/Asturian). This locality was intensively sampled by the amateur paleontologist Michael Sowiak and one of us (AL). The type material with Pal numbers is deposited in the Museum am Schölerberg (Osnabrück, Germany) and the specimens with prefix F in the accession number are from the collection of M. Sowiak (Glandorf, Germany), later deposited in the Museum am Schölerberg or another public accessible institutional collection^70^.

### Locality and age

Piesberg quarry near Osnabrück (Lower Saxony, Germany) is currently one of few accessible and well sampled Pennsylvanian (Moscovian) localities in Western Europe with an abundance of fossil insects and other arthropods in several horizons of shales above coal seams and one limited laminated shale layer without basal coal seam (e.g., Leipner et al.^70^). Pterygotes are dominated by palaeopteran and polyneopteran insect groups along with isolated records of holometabolans (e.g., Brauckmann and Herd^71,72^; Prokop et al.^41^). The record of palaeodictyopteran imagoes known from Piesberg quarry currently includes 12 described species assigned to 6 families, which makes it one of the most species rich Pennsylvanian localities^36^.

### Light imaging

Fossils were examined under Nikon SMZ 745 T or Zeiss SteREO Discovery.V20 stereomicroscopes. Macrophotography was undertaken using a digital camera Canon EOS 550D (Canon Inc., Tokyo, Japan) coupled with MP-E 65 mm or FE-S 50 mm macro photo lenses, or linked to a Nikon SMZ 745 T microscope illuminated by a Schott spotlight. The photograph of Pal cl 4 was taken with a Canon EOS 450 D and EF-S 60 mm macro photo lens under a film of ethanol. The photographs were processed using the image-editing software Adobe Photoshop CS and those taken at different distances were focus stacked using the stacking software Helicon focus Pro 6.2.2 (Helicon Soft Ltd., Kharkov, Ukraine) or Zerene Stacker (Zerene systems LLC, Richland, U.S.A.). Composed image of the *Idoptilus* sp. was obtained from digital microscope Keyence VHX VH-Z20UR located at the National Museum in Prague.

### Environmental scanning electron microscopy (ESEM)

Scanning electron micrographs were taken using an environmental electron microscope Hitachi S-3700 N (Hitachi Ltd, Chiyoda, Tokyo, Japan) at an accelerating voltage of 15 kV with a turntable sample holder, which is located at the National Museum in Prague.

Abbreviations used for morphological structures are: ca, costal area, ce, cerci, pl, prothoracic lobe; mswp, mesothoracic wing pad; mtwp, metathoracic wing pad; sc, scutum; scl, scutellum. Lacunae of precursor veins: AA—anal anterior vein, CuA/CuP—cubitus anterior/posterior, MA/MP—media anterior/posterior, RA/RP—radius anterior/posterior, ScP—subcosta posterior.

### Reporting summary

Further information on research design is available in the Nature Portfolio Reporting Summary linked to this article.

### Supplementary information

Supplementary Information

Reporting Summary

## Data Availability

The authors declare that all data supporting the findings of this study are available within the article. List of the fossil specimens examined in this study: Pal1242ab, Pal1243, F429ab, F139, F148ab, F246ab, F320, F375ab, F427ab and Pal cl 4. All data are available in the main text, Supplementary Note 1, Supplementary Table 1, and Supplementary Information file provided.

## Materials availability

All specimens in this study were examined from the following institutional collections: Manchester Museum, Manchester, UK (MM); Muséum national d’Histoire naturelle, Paris, France (MNHN); The Natural History Museum, London, UK (NHM) and Museum Am Schölerberg, Osnabrück, Germany (Pal).
