# Author Correction: Thoracic and abdominal outgrowths in early pterygotes: a clue to the common ancestor of winged insects?

**DOI:** 10.1038/s42003-024-05803-8

**Published:** 2024-01-25

**Authors:** Jakub Prokop, Kateřina Rosová, Angelika Leipner, Pavel Sroka

**Affiliations:** 1 Department of Zoology, Faculty of Science, Charles University, Viničná 7, 128 00 Praha 2, Czech Republic; 2 Museum Schölerberg, Klaus-Strick-Weg 10, 49082 Osnabrück, Germany; 3 Institute of Entomology, Biology Centre of the Czech Academy of Sciences, Branišovská 31, 370 05 České Budějovice, Czech Republic

**Keywords:** Palaeontology, Entomology

Correction to: *Communications Biology* 10.1038/s42003-023-05568-6, published online 12 December 2023.

In the Acknowledgements section of this article the grant number relating to Grant Agency of the Czech Republic given for Jakub Prokop was incorrectly given as No. 18-03118S and should have been No. 21-05216S. The original article has been corrected.

