# Supplementary material for: Thoracic and abdominal outgrowths in early pterygotes: a clue to the common ancestor of winged insects?
Source: Commun Biol. 2023 Dec 12;6:1262. doi: 10.1038/s42003-023-05568-6 (PMC10716172; doi:10.1038/s42003-023-05568-6)
Supplement: Supplementary file 1 — Supplementary Information [file 42003_2023_5568_MOESM1_ESM.pdf]

Supplementary Information

Thoracic and abdominal outgrowths in early pterygotes: a clue to the common ancestor of winged insects?

Jakub Prokop[1]* Kateřina Rosová[1] Angelika Leipner[2] & Pavel Sroka[3]

[1]Department of Zoology, Faculty of Science, Charles University, Viničná 7, CZ-128 00 Praha 2, Czech Republic. [2]Museum Am Schölerberg, Klaus-Strick-Weg 10, DE-49082 Osnabrück Germany. [3]Institute of Entomology, Biology Centre of the Czech Academy of Sciences, Branišovská 31, CZ-37005 České Budějovice, Czech Republic.
*e-mail: jprokop@natur.cuni.cz

Including :

Supplementary Note 1
Supplementary Table 1

Supplementary Note 1

List of examined specimens:

Holotype specimen No. Pal1242ab (Clay stone layer above the Dreibänke coal seam) – (previously considered as nymph – „Rochdalia type“ with ovipositor), presumably exuvia due to distortion of metathoracic wing pads, prothoracic winglet lobe without distinctive articulation area – single vein precursor very faint in comparison to wing pads, meso- and metathoracic wing pads with well discernible pattern of lacunae, abdomen with terminalia. Structure of wing pads: WP1: ScP probably reaching RA behind midwing, precursors of RA, MA and CuA all simple, RP with 6 branches, MP with 7 branches, CuP with 4 branches, PCu deeply bifurcated, both probably secondary twigged, A with 6 branches; WP2: veins RA, MA and CuA all simple, RP with 8 branches, MP with 10 branches, CuP with 5 branches, anal area (including PCu) with 2? branches.

Specimen No. F375ab (Clay stone layer above the Dreibänke coal seam) – ?exuvia – prominent prothoracic winglet lobe, meso- and metathoracic wing pads (mesothoracic wing pad with keel and well discernible pattern of lacunae while in metathoracic only posterior part is discernible), abdominal segments I-IV with prominent lateral outgrowths (articulation of wing pads / abdominal outgrowths). Structure of wing pads: WP1: prominent keel, ScP running close to radius, three simple convex precursors of RA, MA, CuA, RP with 5? branches, MP with 6? branches, CuP simple or with two branches, anal area broad with numerous branches pectinated (about 5 branches); WP2: only distal part of wing pad showing the same as WP1, but with reduced costal area, three simple convex precursors of RA, MA, CuA, RP and MP similar to WP1, basal part of wing pad not preserved.

Specimen No. F429ab (Clay stone layer above the Dreibänke coal seam) – tip of one metathoracic wing pad and abdomen with prominent lateral outgrowths, caudal appendages not preserved (only fragments). Structure of wing pad: WP2: apex with discernible ScP running rather close along radius, RP with about 3 branches, convex MA probably simple.

Specimen No. F246ab (Clay stone layer above the Dreibänke coal seam) – exuvia based on distortion of meso- and metathoracic wing pads, thorax distorted with meso- and metathoracic wing pads, abdomen with prominent lateral outgrowths and caudal appendages, faintly preserved structures resembling valvulae of ovipositor discernible between abdominal segments VIII and IX. Structure of wing pads: WP1: ScP running very closely to RA in distal part of the wing, precursors of RA, MA, CuA all simple, RP with 3-4 branches, MP with 5? branches, CuP with number of branches not clearly discernible, broad anal area behind with about 5 branches incl. PCu, prominent widened posterior margin of wing; WP2: wing apex sunken (visibly jutting out of the line of the posterior margin), precursors of RA, MA, CuA all simple, RP with 6-7 branches, MP with about 7 branches, CuP with number of branches, anal area rather broad.

Specimen No. F148ab (Clay stone layer above the Dreibänke coal seam) – dorsoventral aspect, probably exuvia with metathoracic wing pads exposed, very well preserved abdominal lateral outgrowths, caudal appendages well preserved. Structure of wing pad: WP2: veins RA, MA, CuA all simple, RP rather reduced with 2 or max. 3 branches, MP with 4-5 branches, CuP with ?2-3 branches, PCu and anal area rather broad.

Specimen No. F427ab (Clay stone layer above the Dreibänke coal seam) – lateral aspect, presumably mesothoracic wing pad without preserved thoracic connection, but corresponding to the position. Distal part of abdomen showing segments III-X with well discernible caudal appendages and lateral abdominal outgrowths (presumably shortened epiproct and paired cone-shaped paraprocts, small reduced cerci are not well discernible – more distinct under

film layer of ethylalcohol, abdominal segment X with a short ventral protrusion). Structure of wing pad: Basal part of mesothoracic wing pad on one slab while the distal part on the other one. WP2: Costal area broad but not triangular like in other specimens, ScA well discernible convex running towards costal margin, ScP running parallel to RA, convex veinal precursors of MA and CuA discernible, RP and MP with more branches, but impossible to count (MP area seems to be broader over RP), CuP not clearly discernible from anal area, anal area rather broad with numerous veins.

Specimen No. F320 (Clay stone layer above the Dreibänke coal seam) – dorso-lateral aspect, thoracic segments distorted, abdomen with well discernible caudal appendages in form of paraprocts and perhaps also with epiproct, lateral flaps discernible only on some abdominal segments, mesothoracic wing pad with broad costal keel and presumably numerous branches of MP (4-5), otherwise vein precursors not well discernible.

Specimen No. Pal1243 (Clay stone layer above the Dreibänke coal seam) – dorso-lateral aspect, thoracic segments distorted, ?exuvia, abdomen with well discernible caudal appendages in form of paraprocts and perhaps also together with epiproct, lateral flaps not well preserved.

Specimen No. F139 (Clay stone layer above the Dreibänke coal seam) – dorsal aspect, thoracic segments with prothoracic lobe, meso- and metathoracic wing pads and proximal segments of abdomen, lateral outgrowths well discernible, wing pads with only partly discernible pattern of lacunae.

Specimen No. Pal cl4 (lake sediment between the coal seams Mittel and Johannisstein) – larval exuvia in ventral aspect, showing abdominal lateral outgrows, valvular ovipositor between segment VIII and IX and caudal appendages. Branching pattern of wing pads not clearly discernible.

Supplementary Table 1

Branching pattern of wing vein precursors and wing pad dimensions

Specimen no.	**F/H**	**RP**	**MA**	**MP**	**CuA**	**CuP**	**wp length (mm)**	**wp width (mm)**
**Pal1242**	F	6	1	7	1	4	14.4	7.7
	H	8	1	10	1	5	13.5	6.9
**Pal1243**	F	?	?	?	?	?	3.2	1.9
**F139**	F	5-6	1	?	1	?	6.9	3.4
	H	~5	1	?	1	?	6.9	2.9
**F148**	H	2-3	1	4-5	1	2-3	4.6	?
**F246**	F	3-4	1	~5	1	?	9.5	?
	H	6-7	1	7	1	?	?	?
**F320**	F	?	?	4-5	?	?	?	?
**F375**	F	5?	1	6?	1	2-3	6.5	4.6
	H	?	1	?	1	?	6.7	?
**F427**	F?	?	1	?	1	?	?	?
**F429**	H	~3	1	?	?	?	?	?
