# Supplementary material for: Thoracic and abdominal outgrowths in early pterygotes: a clue to the common ancestor of winged insects?
Source: Commun Biol. 2023 Dec 12;6:1262. doi: 10.1038/s42003-023-05568-6 (PMC10716172; doi:10.1038/s42003-023-05568-6)
Supplement: Supplementary file 2 — Reporting Summary [file 42003_2023_5568_MOESM2_ESM.pdf]

nature portfolio

Corresponding author(s): Jakub Prokop

Last updated by author(s): Sep 21, 2023

Reporting Summary

Nature Portfolio wishes to improve the reproducibility of the work that we publish. This form provides structure for consistency and transparency in reporting. For further information on Nature Portfolio policies, see our Editorial Policies and the Editorial Policy Checklist.

Statistics

For all statistical analyses, confirm that the following items are present in the figure legend, table legend, main text, or Methods section.

n/a	Confirmed	
☒	☐	The exact sample size (*n*) for each experimental group/condition, given as a discrete number and unit of measurement
☒	☐	A statement on whether measurements were taken from distinct samples or whether the same sample was measured repeatedly
☒	☐	The statistical test(s) used AND whether they are one- or two-sided<br>*Only common tests should be described solely by name; describe more complex techniques in the Methods section.*
☒	☐	A description of all covariates tested
☒	☐	A description of any assumptions or corrections, such as tests of normality and adjustment for multiple comparisons
☒	☐	A full description of the statistical parameters including central tendency (e.g. means) or other basic estimates (e.g. regression coefficient) AND variation (e.g. standard deviation) or associated estimates of uncertainty (e.g. confidence intervals)
☒	☐	For null hypothesis testing, the test statistic (e.g. *F*, *t*, *r*) with confidence intervals, effect sizes, degrees of freedom and *P* value noted<br>*Give P values as exact values whenever suitable.*
☒	☐	For Bayesian analysis, information on the choice of priors and Markov chain Monte Carlo settings
☒	☐	For hierarchical and complex designs, identification of the appropriate level for tests and full reporting of outcomes
☒	☐	Estimates of effect sizes (e.g. Cohen's *d*, Pearson's *r*), indicating how they were calculated

Our web collection on statistics for biologists contains articles on many of the points above.

Software and code

Policy information about availability of computer code

Data collection	Adobe Photoshop CS6 for image processing. Helicon focus Pro 6.2.2 and Zerene Stacker for focus-stacking of some images.
Data analysis	no software was used

For manuscripts utilizing custom algorithms or software that are central to the research but not yet described in published literature, software must be made available to editors and reviewers. We strongly encourage code deposition in a community repository (e.g. GitHub). See the Nature Portfolio guidelines for submitting code & software for further information.

Data

Policy information about availability of data

All manuscripts must include a data availability statement. This statement should provide the following information, where applicable:

- Accession codes, unique identifiers, or web links for publicly available datasets
- A description of any restrictions on data availability
- For clinical datasets or third party data, please ensure that the statement adheres to our policy

Provide your data availability statement here.

Human research participants

Policy information about studies involving human research participants and Sex and Gender in Research.

Reporting on sex and gender	*Use the terms sex (biological attribute) and gender (shaped by social and cultural circumstances) carefully in order to avoid confusing both terms. Indicate if findings apply to only one sex or gender; describe whether sex and gender were considered in study design whether sex and/or gender was determined based on self-reporting or assigned and methods used. Provide in the source data disaggregated sex and gender data where this information has been collected, and consent has been obtained for sharing of individual-level data; provide overall numbers in this Reporting Summary. Please state if this information has not been collected. Report sex- and gender-based analyses where performed, justify reasons for lack of sex- and gender-based analysis.*
Population characteristics	*Describe the covariate-relevant population characteristics of the human research participants (e.g. age, genotypic information, past and current diagnosis and treatment categories). If you filled out the behavioural & social sciences study design questions and have nothing to add here, write "See above."*
Recruitment	*Describe how participants were recruited. Outline any potential self-selection bias or other biases that may be present and how these are likely to impact results.*
Ethics oversight	*Identify the organization(s) that approved the study protocol.*

Note that full information on the approval of the study protocol must also be provided in the manuscript.

Field-specific reporting

Please select the one below that is the best fit for your research. If you are not sure, read the appropriate sections before making your selection.

☐ Life sciences ☐ Behavioural & social sciences ☒ Ecological, evolutionary & environmental sciences

For a reference copy of the document with all sections, see nature.com/documents/nr-reporting-summary-flat.pdf

Ecological, evolutionary & environmental sciences study design

All studies must disclose on these points even when the disclosure is negative.

Study description	The study involves description and comparison of fossil specimens from the Late Carboniferous of Piesberg in Lower Saxony (Germany). All insect specimens were examined for selected morphological structures on the thorax and abdomen.
Research sample	The samples include new material of larval exuvia (Nos. Pal1242ab, Pal1243, F429ab, F139, F148ab, F246ab, F320, F375ab, F427ab, Pal cl 4) from Osnabrück Formation, Clay stone layer above the Dreibänke coal seam, except Pal cl 4, which was collected from the lake sediment between coal seam Mittel and Johannisstein without underlying coal seam, Piesberg Quarry near Osnabrück, Lower Saxony, Germany. All specimens are available for scientific study in institutional collection.
Sampling strategy	New fossils from Piesberg quarry (52°19′ 4.0″ N, 8°1′10.0″ E) near Osnabrück (Germany) are preserved in black shales. These were splitted with a careful tap of a rock hammer. In some cases the specimens were prepared with a vibrating needle.
Data collection	Data were collected by direct examination of fossils and comparison of selected structures with extant species, as well as from literature relevant to the subject of our study.
Timing and spatial scale	The material from Piesberg quarry was intensively collected over the last decade.
Data exclusions	No data exclusion.
Reproducibility	All newly presented fossil specimens will become available upon publication.
Randomization	No data randomization.
Blinding	No blinding.

Did the study involve field work? ☐ Yes ☒ No

Reporting for specific materials, systems and methods

We require information from authors about some types of materials, experimental systems and methods used in many studies. Here, indicate whether each material, system or method listed is relevant to your study. If you are not sure if a list item applies to your research, read the appropriate section before selecting a response.

Materials & experimental systems

n/a	Involved in the study
☒	☐ Antibodies
☒	☐ Eukaryotic cell lines
☐	☒ Palaeontology and archaeology
☒	☐ Animals and other organisms
☒	☐ Clinical data
☒	☐ Dual use research of concern

Methods

n/a	Involved in the study
☒	☐ ChIP-seq
☒	☐ Flow cytometry
☒	☐ MRI-based neuroimaging

Palaeontology and Archaeology

Specimen provenance	New specimens were collected by Angelika Leipner and Michael Sowiak in Piesberg quarry, Lower Saxony, Germany. The type material with Pal numbers is deposited in the Museum am Schölerberg (Osnabrück, Germany) and the additional specimens with prefix F in the accession number are from the collection of M. Sowiak (Glandorf, Germany), later deposited in the Museum am Schölerberg or another public accessible institutional collection.
Specimen deposition	All specimens in this study are housed in institutional collections with free access to other researchers.
Dating methods	N/A

☐ Tick this box to confirm that the raw and calibrated dates are available in the paper or in Supplementary Information.

Ethics oversight	Charles Univesity, Faculty of Science, Prague, Czech Republic

Note that full information on the approval of the study protocol must also be provided in the manuscript.
